# Investigation of the Effects of Different Curing Methods on the Adhesion Strength of Single-Lap Joints Produced by Bonding 3D-Printed ABS and PLA Plates with Different Epoxy Adhesives

**DOI:** 10.3390/polym17060768

**Published:** 2025-03-14

**Authors:** Muhammed S. Kamer

**Affiliations:** Department of Mechanical Engineering, Faculty of Engineering and Architecture, Kahramanmaras Sutcu Imam University, Kahramanmaras 46040, Turkey; msafakamer@ksu.edu.tr

**Keywords:** 3D printing, ABS, PLA, curing method, adhesion strength, failure modes, single-lap joint

## Abstract

When bonding 3D-printed polymer products produced by the FFF method, it is essential to determine the appropriate adhesives and assess the resulting adhesion strength. This study focused on producing SLJ test specimens by bonding 3D-printed ABS and PLA plates using Araldite 2011, Araldite 2015-1, and Araldite 2021-1 adhesives. The bonding processes involved various curing methods: without oven (WO), 40 °C for 3 h, 40 °C for 16 h, 60 °C for 2 h, and 80 °C for 1 h. This study aimed to investigate the impact of different adhesives and curing conditions on the bonding strengths of the SLJ test specimens made from 3D-printed ABS and PLA plates. The results showed that the highest tensile strength and elongation at break values for both the ABS and PLA SLJs were achieved in specimens cured at 80 °C for 1 h, irrespective of the adhesive used. Specifically, the maximum tensile force values for the ABS SLJs ranged from 1386.26 N to 1743.98 N, while for the PLA SLJs, the values ranged from 2690.48 N to 3374.77 N. Additionally, the elongation at break values for the ABS SLJs varied from 2.463 mm to 3.485 mm, and for the PLA SLJs, they ranged from 3.260 mm to 4.300 mm.

## 1. Introduction

Three-dimensional (3D) printers that work with the Fused Filament Fabrication (FFF) method are currently used in areas such as product prototyping, final products, etc. Acrylonitrile Butadiene Styrene (ABS) and Polylactic Acid (PLA) filaments are commonly used in FFF 3D printers. It can be said that products produced with ABS materials exhibit flexible behavior, while products produced with PLA materials exhibit brittle behavior. For the products produced with a 3D printer to be used as final products, the mechanical properties of the products must be known. Many studies have been conducted in the literature to determine the mechanical properties of 3D-printed products using ABS [1,2] and PLA [3,4,5] filaments. When using 3D-printed ABS and PLA materials as prototypes or final products, applications that require bonding are encountered. While the process of joining by bonding may be planned in the design of the product, it may also be possible to repair the product by bonding it due to any reason, such as breakage, deformation, etc., during use. In such cases, it is important to determine which adhesive and under what conditions can higher bonding strength be provided when 3D-printed polymer materials are bonded. The single-lap joint (SLJ) test method is a frequently used method for determining the bonding strength and the adhesion ability of the adhesive to the surface of the bonded material when various materials are bonded with different adhesives. With the SLJ test method, the failure modes of the adhesively bonded test samples after the test are also determined [6,7,8].

This paragraph provides an overview of studies in the literature that have utilized the SLJ test method. Saraç et al. [9] investigated the fatigue strength of SLJs bonded with nanoparticle-reinforced adhesives. For this purpose, AISI 304 steel plates were bonded at different overlap lengths (20, 25, and 30 mm) with the DP460 two-component epoxy adhesive produced by 3M Company, to which nano-Al_2_O_3_, nano-TiO_2_, and nano-SiO_2_ powders were added at different ratios (0, 4, and 6%). They revealed that the average failure load in nanoparticle-reinforced adhesive joints was significantly increased. Saraç et al. [10] bonded 2 mm thick AISI 304 steel plates with an overlap length of 20 mm using the DP460 two-component epoxy adhesive, to which they added Al_2_O_3_, TiO_2_, and SiO_2_ nanoparticles with average sizes of 15~20 nm at different rates (0, 2, 4, and 6%). They investigated the static and fatigue strengths of the SLJs they produced in this way. They determined that the highest average failure load was achieved when 4 wt% Al_2_O_3_ nanoparticles were added to the epoxy adhesive. Ozel et al. [11] designed SLJs by joining steel plates of different thicknesses (1.6, 3, and 8 mm) with two different adhesives (AV 119 and SBT 9245) and different overlap lengths (12.5, 25, 50, and 100 mm). They examined the changes in the strength of the joints by exposing their designs to bending moments in a computer environment using the finite element method. They determined that adhesive thickness significantly affects the strength of the joint.

This paragraph outlines studies from the literature that utilize the SLJ test method with 3D-printed ABS and PLA plates. Hiremath et al. [12] investigated the effects of 3D printing directions (0°, 45°, and 90°) and adding graphene nanoparticles to the epoxy adhesive at different ratios (0.25, 0.5, 0.75, and 1 wt%) on the tensile and shear mechanical properties of SLJ test samples produced with 3D-printed PLA plates using the FFF method. They determined that the 0° printing direction had the highest load carrying and shear strength compared to the 45° and 90° printing directions. They also found that adding graphene nanoparticles to the epoxy adhesive greatly increased the bonding strength. Cavalcanti et al. [13] laminated natural (jute and curau’a) and synthetic (glass) fiber reinforcement fabrics to the top and bottom surfaces of 3D-printed ABS and PLA plates using epoxy resin by compression molding. They fabricated SLJ test specimens using 3D-printed plates and 3D-printed plates fabricated with fiber reinforcement. They determined that the mechanical performance of SLJ test specimens produced by adding fiber reinforcement to 3D-printed plates was higher than SLJ test specimens produced using only 3D-printed plates. Oz and Ozturk [14] produced SLJ test specimens by bonding 3D-printed PLA plates with the DP-8005 acrylic adhesive. They investigated the effects of changing the 3D printing angle (0°, 45°, and 90°), adherend thickness (2, 4, and 6 mm), and overlap length (12.7 and 25.4 mm) on the mechanical properties of SLJ test specimens. They determined that the printing angle and adherend thickness had a more significant effect on the adhesion strength in the SLJs with an overlap length of 25.4 mm. Bechtel et al. [15] produced SLJ test specimens by bonding 3D-printed ABS, PETG, and PLA plates to 2 mm thick EN AW-6082-T6 aluminum plates using the Henkel brand Loctite EA3430 epoxy adhesive. They investigated the temperature dependence of polymer melt rheology, wetting behavior, aging effect, and SLJ shear strength variables in the test specimens they produced. They determined that PETG was the most suitable 3D-printed material for creating structural connections. As can be seen, different adhesives have been used in the literature to determine the SLJ adhesion strength of 3D-printed plates. In this study, three different two-component epoxy adhesives, namely Huntsman brand Araldite 2011, Araldite 2015-1, and Araldite 2021-1, were used to bond 3D-printed ABS and PLA plates.

This paragraph outlines studies from the literature that have employed the Araldite 2011 epoxy adhesive. Ejaz et al. [16] investigated the effects of adding reduced graphene oxide (RGO) to the Araldite 2011 epoxy adhesive at the rates of 0.25, 0.5, 0.75, and 1 wt% on the mechanical properties of single- and dual-lap joints. They used aluminum Al 5083 plates as the adherend. They emphasized that with the addition of 0.25 wt% RGO to the epoxy adhesive, a 9.5% increase in tensile strength and a 128% increase in toughness were observed. Ghasemvand et al. [17] investigated the effects of adhesive defects and their location on the strength and creep behavior of SLJs. They bonded aluminum Al 6060-T5 plates together by creating defects on various sides of the Araldite 2011 epoxy adhesive layer. They applied creep tests at three different temperatures (23, 45, and 55 °C) and tensile tests to the SLJ test samples they produced in this way. They determined that the presence of a defect in the adhesive layer caused the greatest strength reduction in the imperfect joint where the defect was in the middle and on both sides. They found that creep displacement increased with increasing temperature. Gültekin and Yazıcı [18] developed nanocomposite adhesives by mixing hexagonal boron nitride (hBN) and hexagonal boron carbide (hB_4_C) nanoparticles at 0.5, 1, 2, and 3 wt% into Araldite 2011 and MGS-LR285 epoxy adhesives. They produced SLJ test specimens by bonding plain-woven carbon-fiber-fabric-reinforced composite plates together using the adhesives they developed. They determined that there was a significant increase in the failure load of the bonded joints with functionalized hBN and hB_4_C doped nanocomposite adhesives. Erbayrak [19] produced SLJ test specimens by bonding woven carbon-fiber-reinforced epoxy (CFRE) laminate plates, plain-woven glass-fiber-reinforced epoxy (GFRE) laminate plates, and hybrid composite laminate (HCGFRE) plates formed by stacking carbon and glass composite laminates using Araldite 2011 and Araldite 138M/HV998 epoxy adhesives. Low-speed impact tests with 40 J impact energy were applied to the SLJ test specimens produced with two different epoxy adhesives using similar or different plates. The highest reaction force was in the specimens produced by bonding HCGFRE and GFRE plates with the Araldite 2011 epoxy adhesive, and the lowest reaction force was in the specimens produced by bonding GFRE and CFRE plates with the Araldite138M/HV998 epoxy adhesive.

A selection of studies in the literature conducted using the Araldite 2015-1 epoxy adhesive is summarized here. Liu et al. [20] produced SLJ test specimens by bonding basalt-fiber-reinforced polymer (BFRP) plates to aluminum Al 5052 plates using Araldite 2012 and Araldite 2015 adhesives. They tested the specimens they produced by keeping them in different immersion solutions (salt sprays under 80 °C, 3.5% NaCl environment; 80 °C, 5% NaCl environment; and pure water environment). They determined that joints formed with the Araldite 2012 adhesive had higher tensile strength and greater ductility in the 5% NaCl environment. Zamani et al. [21] investigated the fatigue behavior of SLJs formed by bonding Al 2024-T3 and GFRP plates together by adding nanographene and nanosilica particles in different mass ratios to the Araldite 2015 epoxy adhesive under four-point bending load. They determined that the average static failure load of the nanographene-reinforced SLJs was higher than that of the 0.5 and 1 wt% nanosilica-reinforced joints. Jairaja and Naik [22] fabricated SLJ test specimens with CFRP and AA6082-T651 plates using a single or double adhesive with Araldite 2015 and AV138 adhesives. In the case of the dual adhesive, the ductile adhesive Araldite 2015 was used at the ends of the overlap due to its high shear and peel strength, while the brittle adhesive AV138 was used in the middle of the overlap in different sizes. They determined that the use of dual adhesives increased the bonding strength.

This paragraph provides an overview of studies in the literature that have employed the Araldite 2021-1 epoxy adhesive. Meulman et al. [23] developed a method to determine viscoelastic creep crack growth and how it affects the mechanical behavior of an adhesive and to obtain the average crack growth rate as a function of the energy release rate. They bonded aluminum Al 7075-T6 plates together using the Araldite 2021-1 epoxy adhesive and thus produced mode I test samples. They designed a cylinder-wedge-driven test rig using a cylinder wedge to test mode I double cantilever beam (DCB)-like specimens. They determined that applying a relatively low energy release rate would lead to creep crack growth compared to the fracture toughness of Araldite 2021-1 determined by quasi-static tests. Manohar et al. [24] investigated the effects of parameters such as different overlap lengths, loading conditions, pretreatment processes, and processing conditions of epoxies on the bonding of carbon-fiber-reinforced polymer (CFRP) materials. In their work, they used Araldite 2021 cold-cure epoxy, F246 hardened acrylic, and ESP110 thermosetting hardened epoxy adhesives. They determined that the ESP110 epoxy adhesive has higher strength compared to the other adhesives. They revealed that the maximum strength of the joint depends on the pretreatment, thickness, and overlap of the joint. Cabello et al. [25] developed an analytical model based on the elastic foundation beam theory for DCB-connected specimens bonded with flexible or rigid adhesives. They validated the model they developed with DCB tests using flexible Silkron-H100 and rigid Araldite 2021 adhesives. They determined that the developed model is particularly suitable when flexible and/or thick adhesives are used.

In this study, SLJ test specimens were produced using 3D-printed ABS and PLA plates. Three different two-component epoxy adhesives, namely Araldite 2011, Araldite 2015-1, and Araldite 2021-1, were used to bond the 3D-printed plates. Five different curing methods were applied in the bonding processes, including WO, 40 °C-3 h, 40 °C-16 h, 60 °C-2 h, and 80 °C-1 h. The SLJ test specimens were produced in 30 different cases using two different 3D-printed materials (ABS and PLA), three different two-component epoxy adhesives (Araldite 2011, Araldite 2015-1, and Araldite 2021-1), and five different curing methods (WO, 40 °C-3 h, 40 °C-16 h, 60 °C-2 h, and 80 °C-1 h). Tensile tests were applied to the produced SLJ test specimens. The effects of different two-component epoxy adhesives and different curing methods on the bonding strengths of the SLJ test specimens produced using 3D-printed ABS and PLA plates were investigated.

## 2. Materials and Methods

In this study, polymer plates with a test area of 5 mm thickness and 25 mm width were produced with an Ultimaker S5 3D printer (Ultimaker B.V., Utrecht, The Netherlands). Ultimaker ABS orange (Ultimaker B.V., Utrecht, The Netherlands) and Ultimaker PLA (Ultimaker B.V., Utrecht, The Netherlands) pearl white filaments with a diameter of 2.85 mm were used in the production of polymer plates. The parameters specified in Table 1 were used in the production of polymer plates with a 3D printer. For parameters other than those given in Table 1, the default settings were used in the Cura v4.8.0 CAM program when the Ultimaker S5 3D printer was selected.

**Figure 1:**
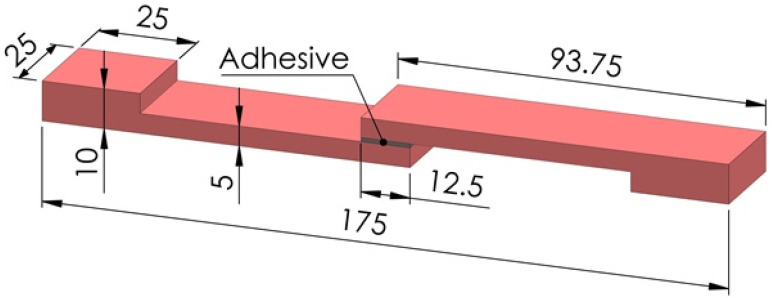
SLJ test specimen dimensions.

**Figure 2:**
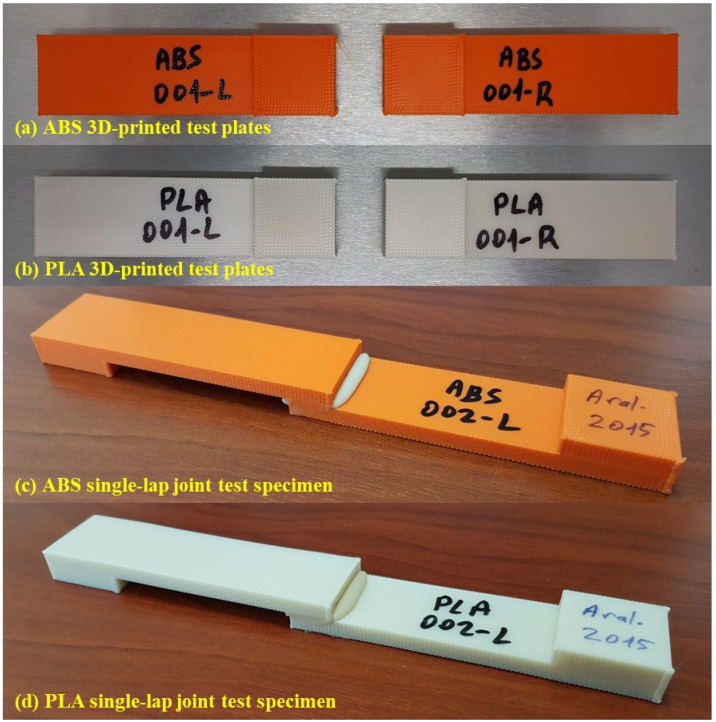
Polymer plate and test specimen images.

The dimensions shown in Figure 1 were used in the production of polymer plates with a 3D printer. Both plates seen in the figure were produced in a single step with a 3D printer, independently of each other. Images of polymer plates produced with a 3D printer using ABS and PLA filaments are shown in Figure 2a and Figure 2b, respectively. The masses, hardness, and surface roughness of the produced polymer plates were measured. A Kern PLS 6200-2A (KERN GmbH, Balingen, Germany) precision balance was used to measure the masses of the polymer plates. A Mitech MH210 (Mitech Co. Ltd., Beijing, China) portable hardness tester was used to measure the hardness values of polymer plates. Hardness measurements were carried out from the upper surfaces of the polymer plates with a Shore D probe, and average values were determined by taking measurements from five different areas of each plate. In hardness measurements, measurements were carried out by selecting the “steel and cast steel” material type in the hardness device. Shore D hardness measurements were carried out on a steel block with a hardness of 786 HLD, 90 mm in diameter, and 56 mm in height. The arithmetic average surface roughness values of the polymer plates were measured with the a Jenoptik Hommel-Etamic W5 (Jenoptik Hommel-Etamic, Villingen-Schwenningen, Germany) surface roughness measuring device. Surface roughness measurements were carried out using the parameters of 4.8 mm measurement length, 0.5 mm/s measurement speed, and 0.8 mm wavelength. Surface roughness measurements were carried out from the top surfaces of the polymer plates parallel to the tensile test direction. Average values were determined by taking measurements from three different areas of each plate.

Test specimens with SLJs were produced by applying an adhesive to an area of 12.5 mm × 25 mm [26,27,28], as shown in Figure 1, with the upper surfaces of the 3D-printed polymer plates corresponding to each other. Images of SLJ test specimens produced using ABS and PLA polymer plates are shown in Figure 2c,d, respectively. Before the bonding process, the polymer plates were washed with detergent water, rinsed, and left to dry. The 3D-printed polymer plates did not undergo any other chemical or mechanical processes. The test plates were not touched with bare hands from the washing process until after the bonding process.

**Figure 3:**
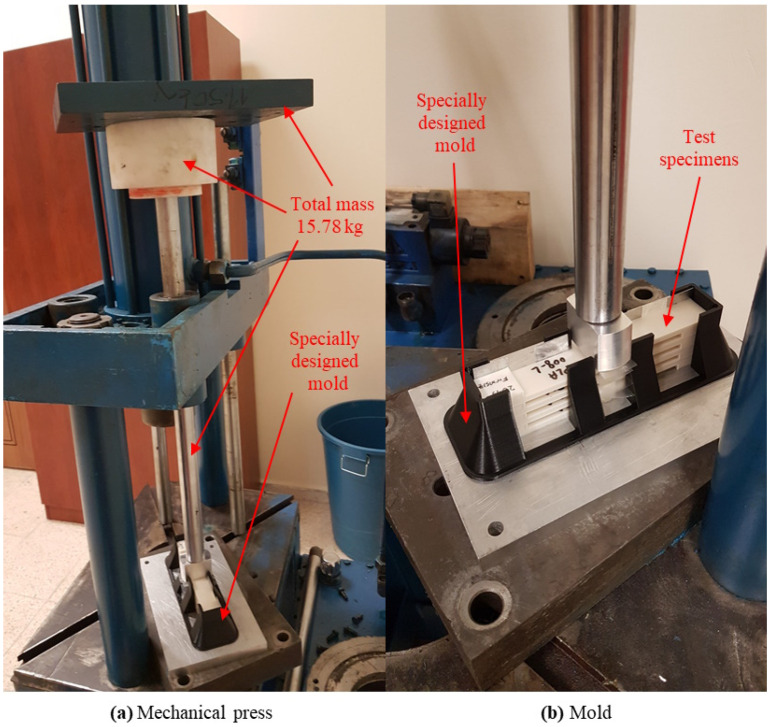
Mechanical press images.

Three different two-component epoxy adhesives, namely Huntsman brand (Huntsman Corporation, The Woodlands, TX, USA) Araldite 2011, Araldite 2015-1, and Araldite 2021-1, were used to produce the SLJ test specimens using 3D-printed ABS and PLA polymer plates. In this way, the adhesion strengths of the different epoxy adhesives on the ABS and PLA polymer plates were investigated. In the manufacturer catalogs of the epoxy adhesives used in this study, an ABS polymer material was also tested, and a 12.5 mm × 25 mm bonding area and a 40 °C-16 h curing method were used in the tests [29,30,31]. The properties of the epoxy adhesives used in the production of the test specimens are given in Table 2. The SLJ test specimens were prepared by applying an epoxy adhesive to a 12.5 mm × 25 mm area on the upper surfaces of the 3D-printed polymer plates corresponding to each other and were kept in a mechanical press at room temperature for one day with a pressure of 0.5 MPa [32,33,34] applied to the bonding surfaces. The image of the mechanical press mechanism used in the bonding process is shown in Figure 3a. To produce test specimens with repeatable dimensions during the bonding process, the test specimens were kept in a mechanical press in a specially designed mold (Figure 3b).

**Figure 4:**
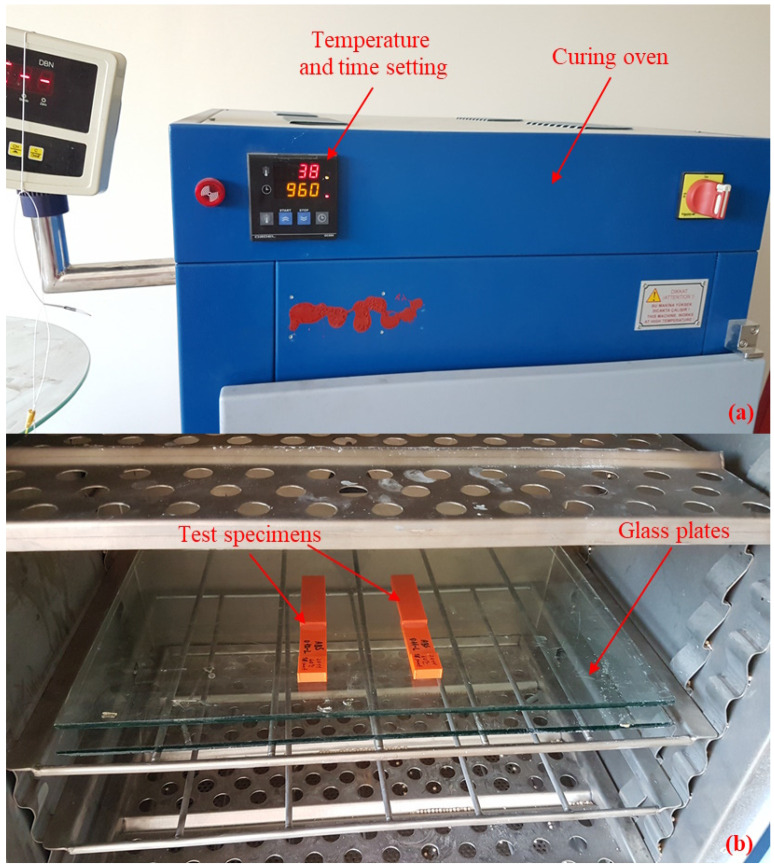
Curing oven images: (**a**) Curing oven exterior view; (**b**) Curing oven interior view.

In the literature, many different curing methods have been used in epoxy adhesive curing, including room temperature [19,37,38], 40 °C-26 h [7], 60 °C-35 min [39], 60 °C-75 min [18], 60 °C-2 h [13,40], 60 °C-16 h [23], 65 °C-35 min [41], 67 °C-30 min [42], 70 °C-30 min [43], 70 °C-2 h [43], 70 °C-12 h [21,44], 80 °C-50 min [18], 80 °C-2 h [45], 120 °C-45 min [46], 120 °C-1 h [28], 120 °C-2 h [32], 150 °C-40 min [27,34], etc. As the curing temperature increases, the curing time will shorten, and there may be a decrease in mechanical properties in materials exposed to higher temperatures than necessary [47]. The point to be considered here was to determine the method that will shorten the curing time without causing a decrease in the mechanical properties of the adherend and adhesive. The SLJ test specimens were produced with five different curing methods, namely WO, 40 °C-3 h, 40 °C-16 h, 60 °C-2 h, and 80 °C-1 h, after mechanical pressing. In the WO production, the test specimens were kept at room temperature for one day after being removed from the mechanical press, and then the tensile test was applied to the specimens. In the with oven production, the test specimens were kept in the oven after being removed from the mechanical press to shorten the curing time of the epoxy adhesive (Figure 4a). To prevent any planar distortion in the test specimens during the oven curing process, the test specimens were placed between two 6 mm thick glass plates and cured (Figure 4b). In the oven process, test specimens were produced using four different temperature–time combinations: 40 °C-3 h, 40 °C-16 h, 60 °C-2 h, and 80 °C-1 h. After the oven process, tensile tests were applied to the test specimens. In this way, the effects of five different curing methods, namely WO, 40 °C-3 h, 40 °C-16 h, 60 °C-2 h, and 80 °C-1 h, on the adhesion strength of epoxy adhesives on ABS and PLA polymer plates were investigated.

**Figure 5:**
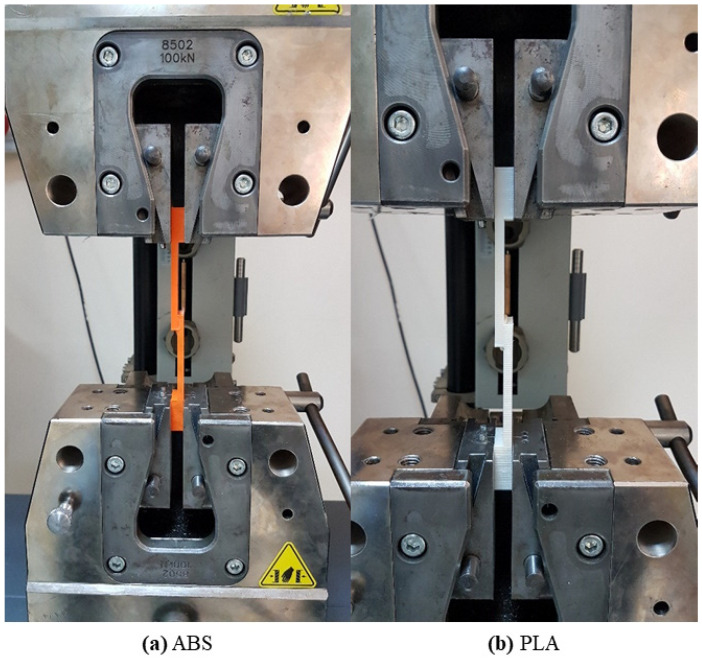
Tensile test images.

The SLJ test specimens were produced in 30 different cases by bonding two different 3D-printed materials (ABS and PLA) using three different two-component epoxy adhesives (Araldite 2011, Araldite 2015-1, and Araldite 2021-1) and five different curing methods (WO, 40 °C-3 h, 40 °C-16 h, 60 °C-2 h, and 80 °C-1 h). Details of the combinations used in the production of test specimens are given in Table 3. A tensile test was applied to the test samples (without oven and with oven), whose production processes were completed after 24 h. Tensile tests were applied to the produced SLJ test specimens according to the ASTM D3163-01 standard [48]. Tensile tests were performed with a Zwick/Roell Z100 tensile testing machine (ZwickRoell Group, Ulm, Germany) at a speed of 1 mm/min [40,49,50]. In the tensile tests, the test specimens were fixed using 100 kN Zwick wedge grips [51]. In the tensile tests, displacement was measured from the AC servo motor system (Danaher Motion GmbH, Düsseldorf, Germany) with a positioning repeatability of ±2.0 μm [52]. In the tensile tests, the force was measured with a GTM brand K series 100 kN load cell (GTM Testing and Metrology GmbH, Bickenbach, Germany) with an error rate of ±0.4% [53]. The tensile tests were repeated three times for each parameter. The graphs that can be shown as averages for each parameter were selected based on the results of the tensile tests. One image from each of the tensile tests of the SLJ test specimens prepared with ABS and PLA polymer plates is shown in Figure 5. Using a Nikon SMZ 1500 microscope (Nikon Corporation, Shinagawa, Tokyo, Japan) with a camera, images of the bonding areas of the broken test specimens after the tensile test were taken. Using these images, the failure modes of the SLJ test specimens were determined.

## 3. Results and Discussion

The mass values of the 3D-printed ABS plates used in the production of the SLJ test specimens ranged from 16.35 to 16.74 g, the hardness values ranged from 68.1 to 69.2 HS, and the arithmetic average roughness values ranged from 3.495 to 5.572 µm. The mass values of the 3D-printed PLA plates used in the production of the SLJ test specimens ranged from 17.57 to 17.89 g, the hardness values ranged from 67.8 to 71.2 HS, and the arithmetic average roughness values ranged from 3.324 to 6.037 µm.

**Figure 6:**
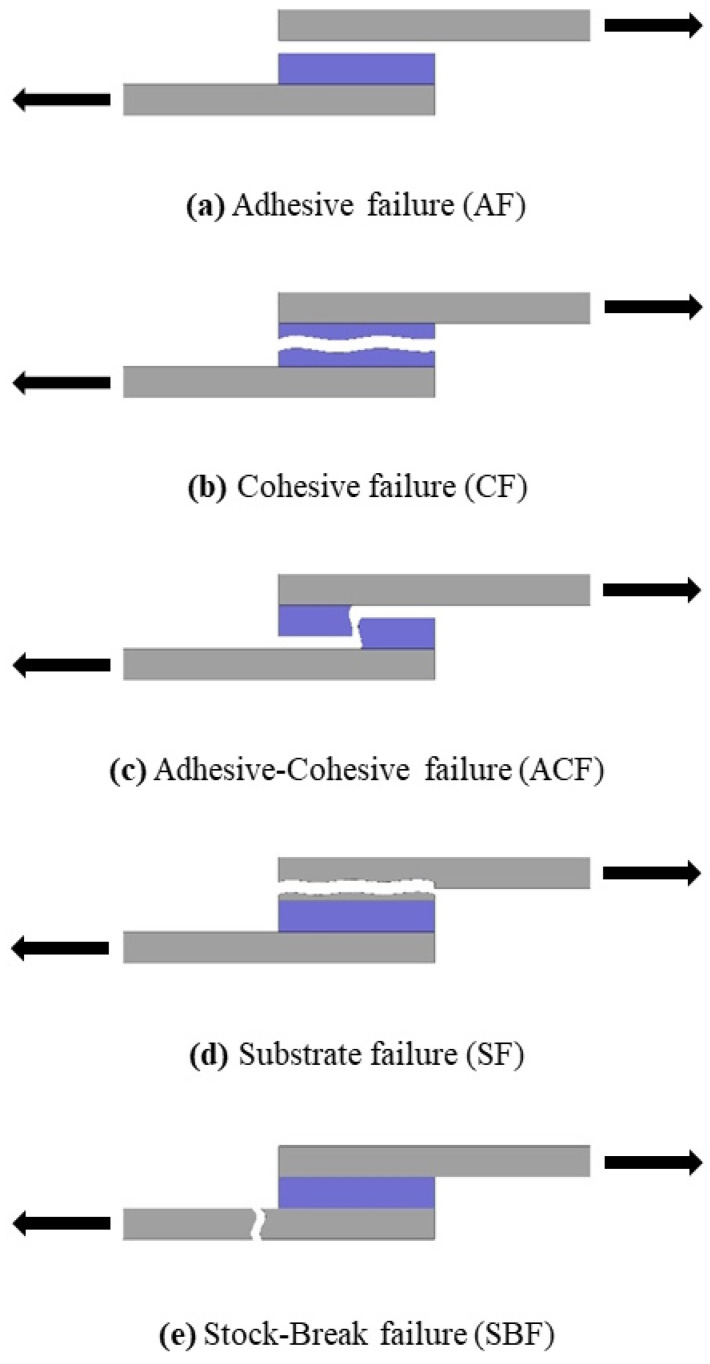
Failure modes of adhesively bonded SLJs.

Different failure modes may occur in tensile tests on adhesively bonded SLJs (Figure 6) [54,55,56]. Adhesive failure (AF) is the complete separation of the adhesive from the surface as a result of the adhesive having difficulty adhering to the plate surface (Figure 6a). Cohesive failure (CF) is when the adhesive adheres to the plate surface and separates as a result of shear stresses in the tensile direction, with some adhesive remaining on the entire bonding surfaces of both plates (Figure 6b). Adhesive–cohesive failure (ACF) is when the adhesive adheres to the surface of the plate, and as a result of the shear stresses in the tensile direction, the adhesive separates transversely from the plate and separates with some adhesive remaining on both plates (Figure 6c). Substrate failure (SF) is a layer separation that occurs on one or both of the plates as a result of the adhesive adhering well to the surfaces of both plates (Figure 6d). Stock-break failure (SBF) is the rupture that occurs in the cross-sectional area of one or both of the plates subjected to tensile stress as a result of the adhesive adhering well to the surfaces of both plates (Figure 6e).

**Figure 7:**
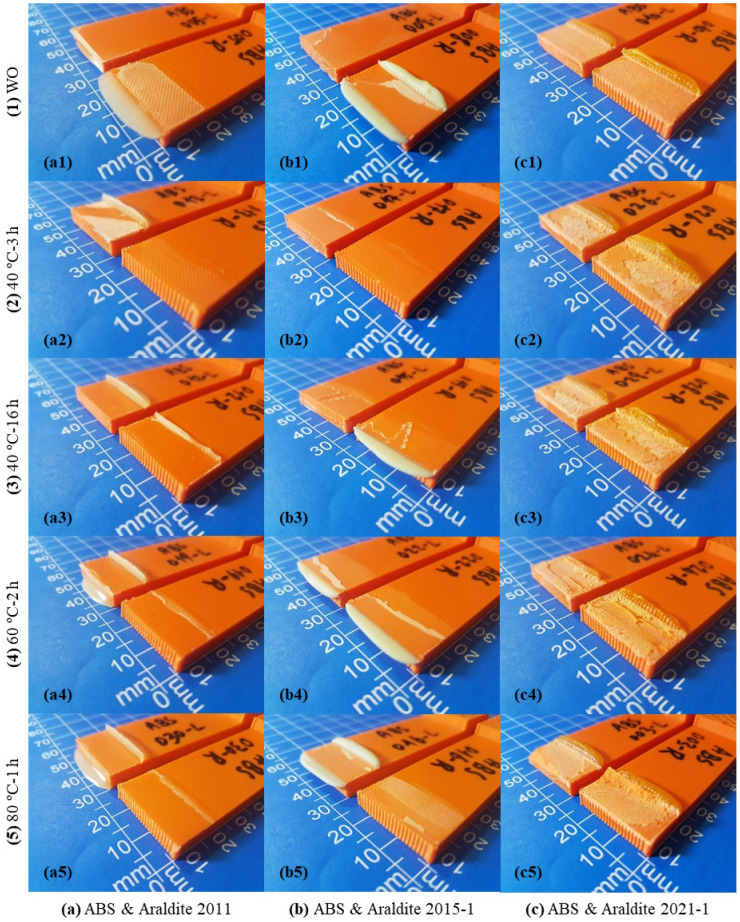
Images of SLJ test specimens made of ABS material after the tensile test.

The images of SLJ test specimens in 15 different cases, which were formed by bonding 3D-printed ABS plates with three different two-component epoxy adhesives (Araldite 2011, Araldite 2015-1, and Araldite 2021-1) using five different curing methods (WO, 40 °C-3 h, 40 °C-16 h, 60 °C-2 h, and 80 °C-1 h), after the tensile test are shown in Figure 7. In the case of bonding the 3D-printed ABS plates with the Araldite 2011 epoxy adhesive, it is seen that the adhesive has difficulty in adhering to the surface in all curing methods (Figure 7a). In the WO and 40 °C-3 h curing methods, it is seen that the adhesive is separated from both plates, and AF occurs (Figure 7(a1,a2)). In the other three curing methods, the adhesive is seen to separate from one plate (Figure 7(a3–a5)). In the case of bonding 3D-printed ABS plates with the Araldite 2015-1 epoxy adhesive, it is seen that the adhesive is generally separated from one plate in the WO, 40 °C-3 h, and 40 °C-16 h curing methods (Figure 7(b1–b3)). In the other two curing methods, it is seen that the rupture occurs when the adhesive separates transversely from the plate and clings to some extent on both plates (Figure 7(b4,b5)). In the case of bonding 3D-printed ABS plates with the Araldite 2021-1 epoxy adhesive, it is seen that CF occurs with some adhesive remaining on both plates in the WO curing method (Figure 7(c1)). In all other curing methods, separation of the layers in the bonding area of the 3D-printed ABS plates is observed (Figure 7(c2–c5)).

**Figure 8:**
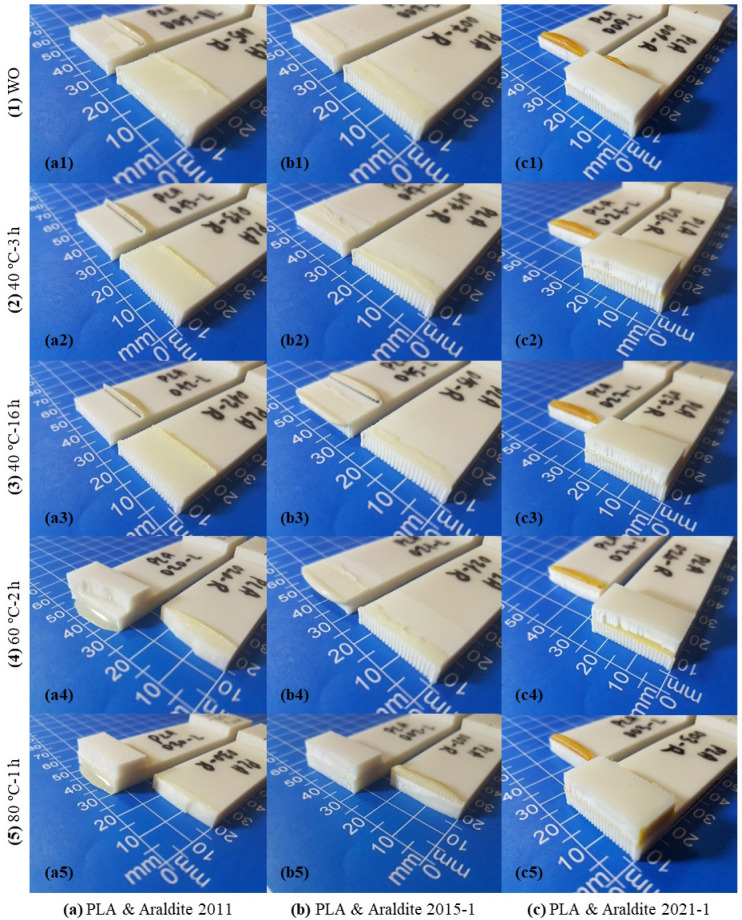
Images of SLJ test specimens made of PLA material after the tensile test.

The images of the SLJ test specimens in 15 different cases, which were formed by bonding 3D-printed PLA plates with three different two-component epoxy adhesives (Araldite 2011, Araldite 2015-1, and Araldite 2021-1) using five different curing methods (WO, 40 °C-3 h, 40 °C-16 h, 60 °C-2 h, and 80 °C-1 h), after the tensile test are shown in Figure 8. In the case of bonding the 3D-printed PLA plates with the Araldite 2011 epoxy adhesive, it is seen that the adhesive has difficulty in adhering to the surface and generally separates from the plate in the WO, 40 °C-3 h, and 40 °C-16 h curing methods (Figure 8(a1–a3)). In the other two curing methods, it is seen that there is no rupture in the bonding area, while SBF occurs in the PLA plate (Figure 8(a4,a5). In the case of bonding the 3D-printed PLA plates with the Araldite 2015-1 epoxy adhesive, in the WO, 40 °C-3 h, 40 °C-16 h, and 60 °C-2 h curing methods, it is seen that the adhesive is separated transversely from the plate, and rupture occurs with some adhesion on both plates (Figure 8(b1–b4)). In the 80 °C-1 h curing method, it is seen that there is no rupture in the bonding area, and SBF occurs in the PLA plate (Figure 8(b5)). In the case of bonding the 3D-printed PLA plates with the Araldite 2021-1 epoxy adhesive, it is seen that there is no rupture in the bonding area in all curing methods, and SBF occurs in the PLA plate (Figure 8(c1–c5)).

**Figure 9:**
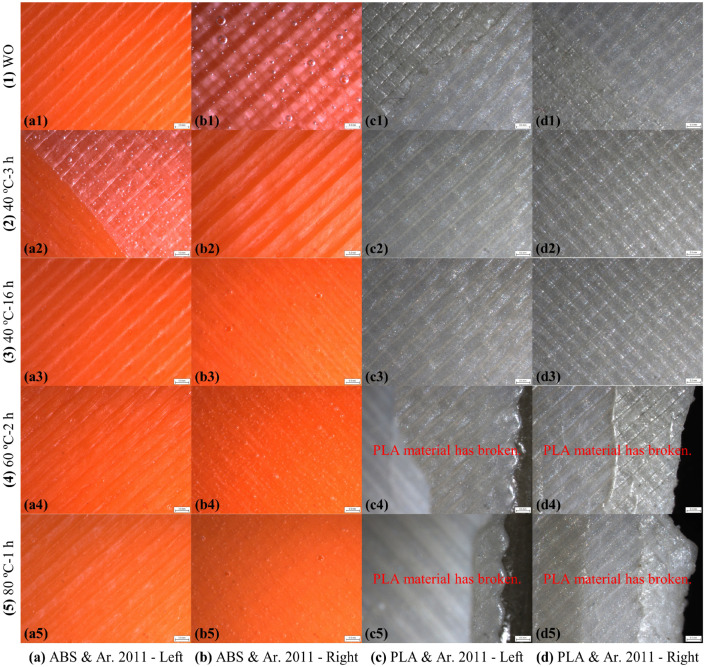
Camera microscope images after tensile testing of bonding areas of ABS and PLA SLJs bonded with Araldite 2011 epoxy adhesive.

The camera microscope images of the left and right plate bonding areas of the ABS and PLA SLJ test specimens bonded with the Araldite 2011 epoxy adhesive after tensile testing are shown in Figure 9. In the SLJ test specimens made with the ABS material, the adhesive could not adhere to the plate surfaces in the WO and 40 °C-3 h curing methods and separated from both plates (left and right plates) in the form of a layer. In the 40 °C-16 h, 60 °C-2 h, and 80 °C-1 h curing methods, the adhesive remained on the ABS plates on the right side and separated. After tensile testing of the ABS SLJ test specimens bonded with the Araldite 2011 epoxy adhesive, the AF failure mode occurred in all five different curing methods. In the SLJ test samples made with the PLA material, the adhesive was separated with some amount remaining on both plates (left and right plates) in the WO curing method. In the 40 °C-3 h and 40 °C-16 h curing methods, the adhesive remained on the PLA plates on the right side and separated. In the 60 °C-2 h and 80 °C-1 h curing methods, no failure occurred in the bonding areas, but rupture occurred from the cross-sectional area exposed to tensile stress in the PLA plates. After the tensile test of the PLA SLJ test specimens bonded with the Araldite 2011 epoxy adhesive, the AF failure mode occurred in the WO, 40 °C-3 h, and 40 °C-16 h curing methods, and the SBF failure mode occurred in the 60 °C-2 h and 80 °C-1 h curing methods. Studies testing the SLJ of 3D-printed PLA plates with the Araldite 2011 epoxy adhesive have also been found in the literature [57]. The failure modes occurring after the tensile test in the ABS and PLA SLJ test specimens bonded with the Araldite 2011 epoxy adhesive are given in Table 4.

**Figure 10:**
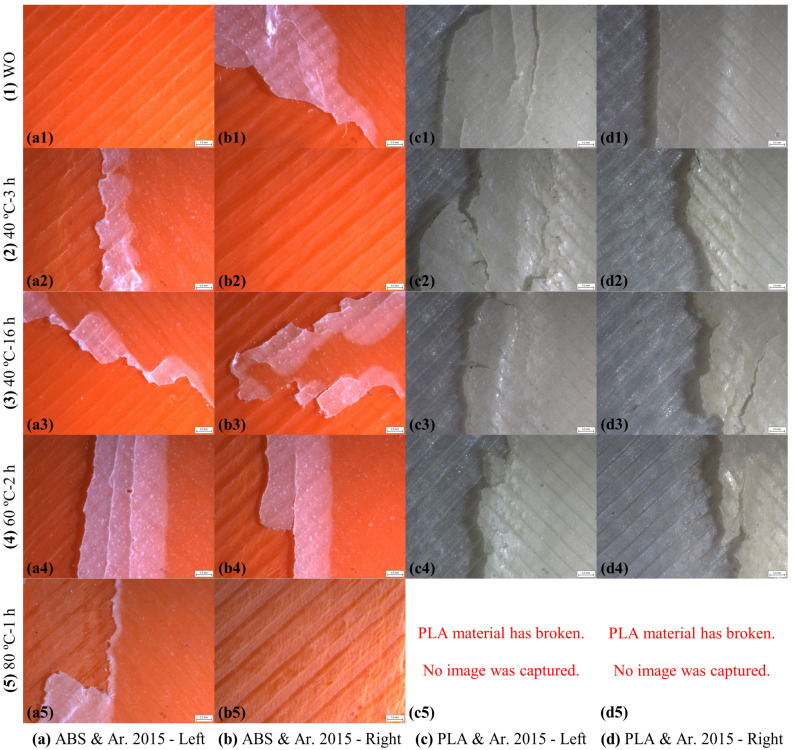
Camera microscope images after tensile testing of bonding areas of ABS and PLA single-lap joints bonded with Araldite 2015-1 epoxy adhesive.

The camera microscope images of the left and right plate bonding areas of the ABS and PLA SLJ test specimens bonded with the Araldite 2015-1 epoxy adhesive after the tensile test are shown in Figure 10. In the test specimens made with the ABS material with a single-lap adhesive bonded connection, the adhesive was separated with some amount remaining on both plates in the WO, 40 °C-3 h, and 40 °C-16 h curing methods. In the SLJ test specimens made with the ABS material, the adhesive was separated on both plates (left and right plates) with some amount remaining on both plates in the WO, 40 °C-3 h, and 40 °C-16 h curing methods. In the 60 °C-2 h and 80 °C-1 h curing methods, the adhesive was separated transversely from the plate, leaving some residue on both plates. The adhesive caused some damage to the ABS plate in the 80 °C-1 h curing method (Figure 10(a5,b5)). After the tensile test of the ABS SLJ test specimens bonded with the Araldite 2015-1 epoxy adhesive, the AF failure mode occurred in the WO, 40 °C-3 h, and 40 °C-16 h curing methods, and the ACF failure mode occurred in the 60 °C-2 h and 80 °C-1 h curing methods. In the SLJ test specimens made with the PLA material, the adhesive was separated on both plates (left and right plates) with some amount remaining on both plates in the WO, 40 °C-3 h, 40 °C-16 h, and 60 °C-2 h curing methods. In the 80 °C-1 h curing method, no failure occurred in the bonding area, and rupture occurred from the cross-sectional area exposed to tensile stress in the PLA plates. After the tensile test of the PLA SLJ test specimens bonded with the Araldite 2015-1 epoxy adhesive, the ACF failure mode occurred in the WO, 40 °C-3 h, 40 °C-16 h, and 60 °C-2 h curing methods, and the SBF failure mode occurred in the 80 °C-1 h curing method. The failure modes occurring after the tensile test on the ABS and PLA SLJ test specimens bonded with the Araldite 2015-1 epoxy adhesive are given in Table 5.

**Figure 11:**
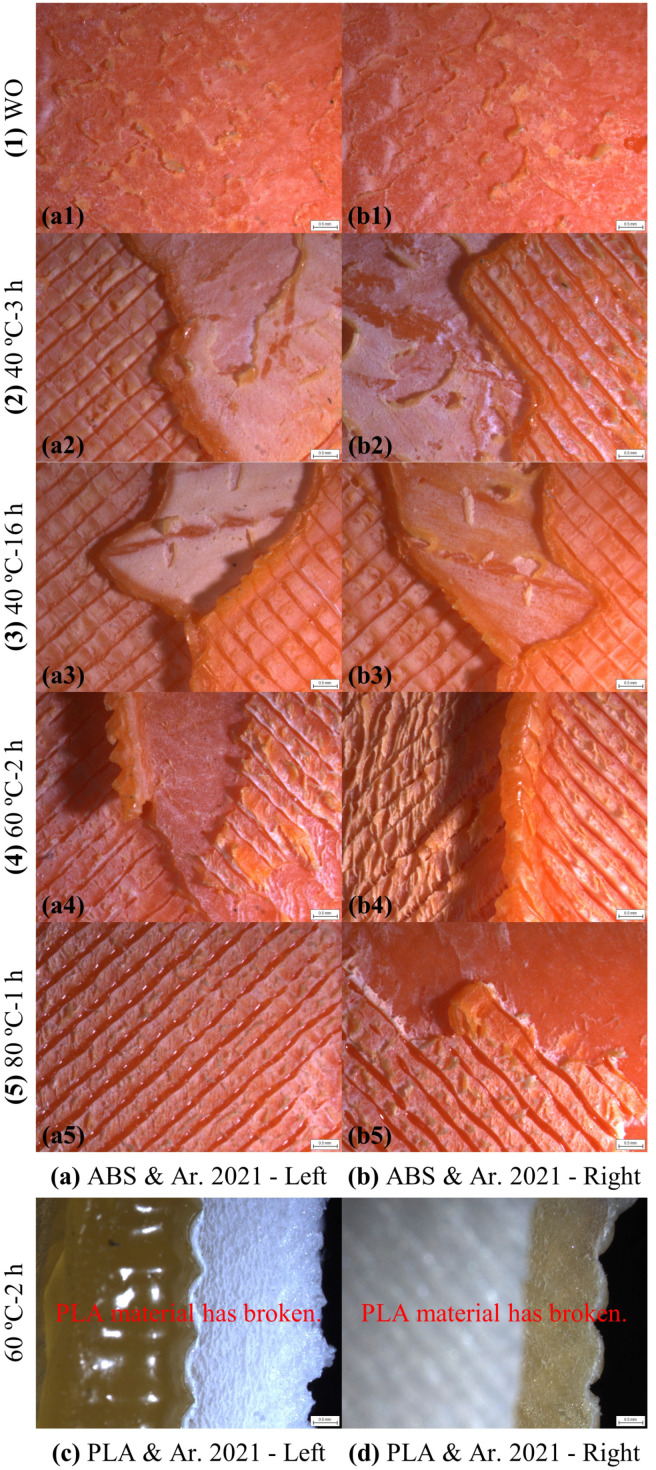
Camera microscope images after tensile testing of bonding areas of ABS and PLA single-lap joints bonded with Araldite 2021-1 epoxy adhesive.

The camera microscope images of the left and right plate bonding areas of the ABS and PLA SLJ test specimens bonded with the Araldite 2021-1 epoxy adhesive after the tensile test are shown in Figure 11. In the SLJ test specimens made with the ABS material, the adhesive was separated with a little bit remaining in the entire bonding surface area on both plates (left and right plates) in the WO curing method. In the 40 °C-3 h, 40 °C-16 h, 60 °C-2 h, and 80 °C-1 h curing methods, there were separations in the layers in the bonding area of the ABS plates. After the tensile test of the ABS SLJ test specimens bonded with the Araldite 2021-1 epoxy adhesive, the CF failure mode occurred in the WO curing method and the SF failure mode occurred in the 40 °C-3 h, 40 °C-16 h, 60 °C-2 h, and 80 °C-1 h curing methods. In the SLJ test specimens made with the PLA material, no failure occurred in the bonding area in all five different curing methods, and rupture occurred from the cross-sectional area exposed to tensile stress in the PLA plates. The Araldite 2021-1 epoxy adhesive caused some damage to the PLA plates (Figure 11c,d). After tensile testing of the PLA SLJ test specimens bonded with the Araldite 2021-1 epoxy adhesive, the SBF failure mode occurred in all five different curing methods. The failure modes of the ABS and PLA SLJ test specimens bonded with the Araldite 2021-1 epoxy adhesive after tensile testing are given in Table 6.

**Figure 12:**
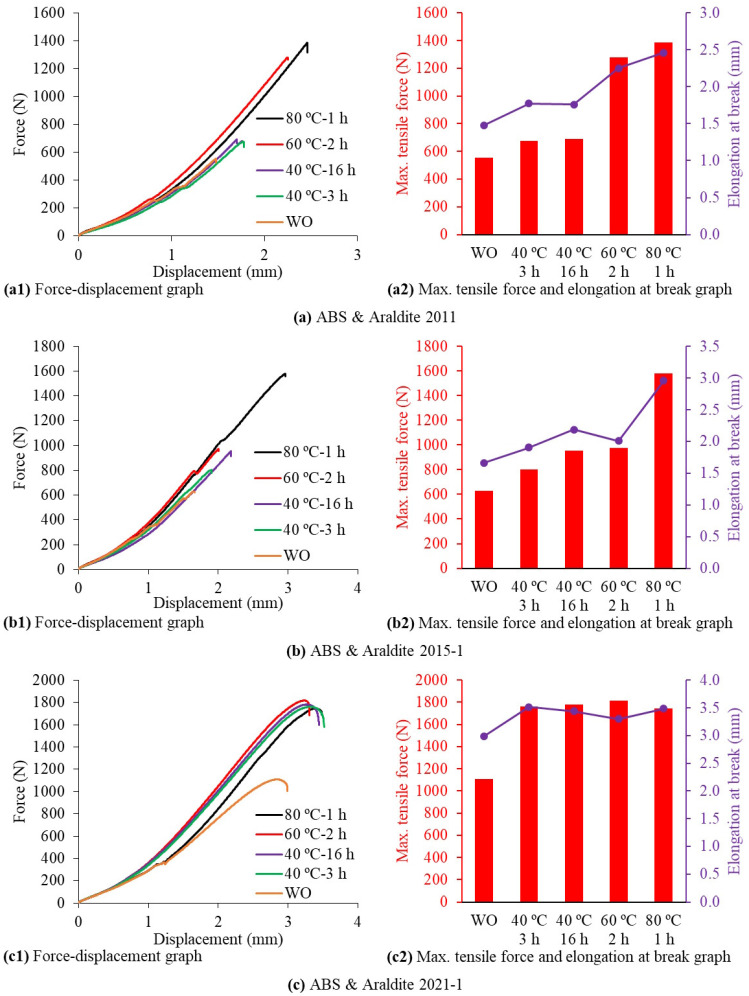
Force–displacement graphs obtained from tensile tests of single-lap joint test specimens made of ABS material.

The force–displacement graphs obtained by applying tensile tests to 15 different cases of SLJ test specimens formed by bonding 3D-printed ABS plates with three different two-component epoxy adhesives via applying five different curing methods are shown in Figure 12. In the case of bonding the ABS plates with the Araldite 2011 epoxy adhesive, there was no significant change between the graphs obtained with the WO, 40 °C-3 h, and 40 °C-16 h curing methods. In the graphs obtained with the 60 °C-2 h and 80 °C-1 h curing methods, there was a 108.4% increase in maximum tensile force and a 40.9% increase in elongation at break compared to the other curing methods. The highest tensile force and highest elongation at break values in the SLJs produced by bonding the ABS plates with the Araldite 2011 epoxy adhesive were in the test specimens produced with the 80 °C-1 h curing method (Figure 12a).

In the case of bonding the ABS plates with the Araldite 2015-1 epoxy adhesive, the maximum tensile force and elongation at break values obtained from the tensile tests increased with increasing curing temperature. The highest tensile force and highest elongation at break values in the SLJs produced by bonding the ABS plates with the Araldite 2015-1 epoxy adhesive were in the test specimens produced with the 80 °C-1 h curing method. In the results obtained with the 80 °C-1 h curing method, there was an 87.9% increase in the maximum tensile force and a 52.5% increase in elongation at break compared to the other four curing methods (Figure 12b).

In the case of bonding the ABS plates with the Araldite 2021-1 epoxy adhesive, the maximum tensile force and elongation at break values obtained with the WO curing method were the lowest values compared to the other four curing methods. There was no significant change between the graphs obtained with the 40 °C-3 h, 40 °C-16 h, 60 °C-2 h, and 80 °C-1 h curing methods. In the graphs obtained with the 40 °C-3 h, 40 °C-16 h, 60 °C-2 h, and 80 °C-1 h curing methods, there was a 60.2% increase in the maximum tensile force and a 14.9% increase in the elongation at break compared to the WO curing method (Figure 12c).

In general, it can be said that the highest tensile force and highest elongation at break values in the SLJs produced by bonding the ABS plates with all three epoxy adhesives are in the test specimens produced with the 80 °C-1 h curing method (Figure 12).

**Figure 13:**
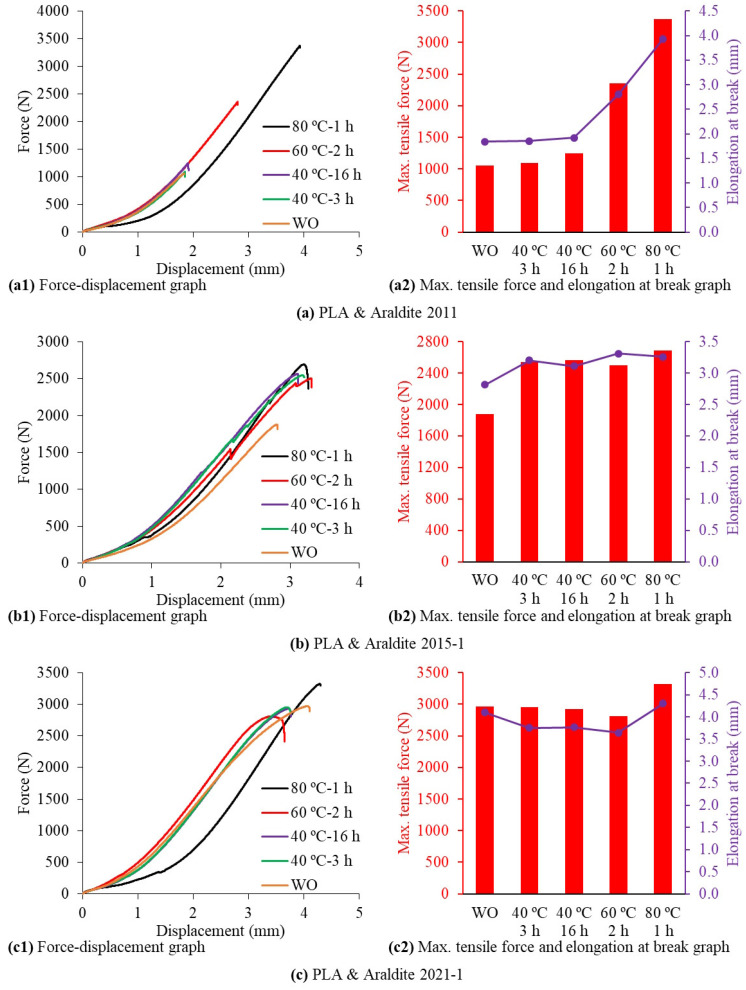
Force–displacement graphs obtained from tensile tests of single-lap joint test specimens made of PLA material.

The force–displacement graphs obtained by applying tensile tests to 15 different cases of SLJ test specimens formed by bonding 3D-printed PLA plates with three different two-component epoxy adhesives via applying five different curing methods are shown in Figure 13. In the case of bonding the PLA plates with the Araldite 2011 epoxy adhesive, there was no significant change between the graphs obtained with the WO, 40 °C-3 h, and 40 °C-16 h curing methods. In the graphs obtained with the 60 °C-2 h and 80 °C-1 h curing methods, the maximum tensile force and elongation at break values gradually increased by at least 40% with the increasing curing temperature compared to other curing methods. The highest tensile force and highest elongation at break values in the SLJs produced by bonding the PLA plates with the Araldite 2011 epoxy adhesive were in the test samples produced with the 80 °C-1 h curing method (Figure 13a).

In the case of bonding the PLA plates with the Araldite 2015-1 epoxy adhesive, the maximum tensile force and elongation at break values obtained with the WO curing method were the lowest values compared to the other four curing methods. There were negligible changes between the graphs obtained with the 40 °C-3 h, 40 °C-16 h, 60 °C-2 h, and 80 °C-1 h curing methods. In the graphs obtained with the 40 °C-3 h, 40 °C-16 h, 60 °C-2 h, and 80 °C-1 h curing methods, there was a 37.3% increase in the maximum tensile force and a 14.5% increase in elongation at break compared to the WO curing method (Figure 13b).

In the case of bonding the PLA plates with the Araldite 2021-1 epoxy adhesive, there was no significant change between the graphs obtained with the WO, 40 °C-3 h, 40 °C-16 h, and 60 °C-2 h curing methods. The highest tensile force and highest elongation at break values in the SLJs produced by bonding the PLA plates with the Araldite 2021-1 epoxy adhesive were in the test specimens produced with the 80 °C-1 h curing method. In the results obtained with the 80 °C-1 h curing method, there was a 14% increase in the maximum tensile force and a 12.7% increase in elongation at break compared to the other four curing methods (Figure 13c).

In general, it can be said that the highest tensile force and highest elongation at break values in the SLJs produced by bonding the PLA plates with all three epoxy adhesives are in the test specimens produced with the 80 °C-1 h curing method (Figure 13).

**Figure 14:**
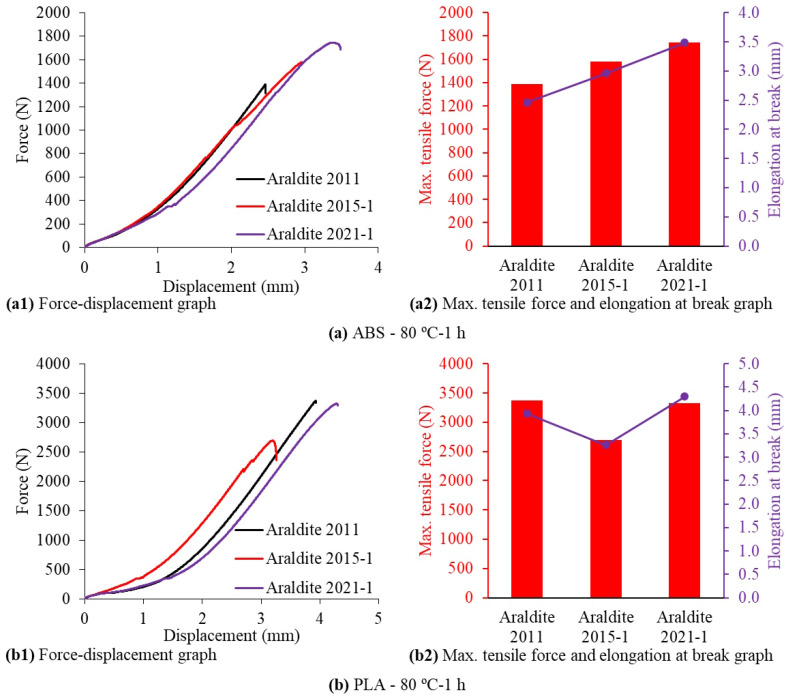
Force–displacement graphs obtained from tensile tests of single-lap joint test specimens made of ABS and PLA materials cured at 80 °C-1 h.

The comparison of force–displacement graphs obtained from the tensile tests of the SLJ test specimens produced by bonding 3D-printed ABS and PLA plates with three different two-component epoxy adhesives using the 80 °C-1 h curing method is shown in Figure 14. When the ABS plates were bonded using the Araldite 2011, Araldite 2015-1, and Araldite 2021-1 epoxy adhesives by applying the 80 °C-1 h curing method, the maximum tensile force and elongation at break values increased, respectively, and this increase exhibited an approximately linear behavior (Figure 14a). When the PLA plates were bonded using the Araldite 2011, Araldite 2015-1, and Araldite 2021-1 epoxy adhesives by applying the 80 °C-1 h curing method, the lowest maximum tensile force and elongation at break values were obtained when the Araldite 2015-1 epoxy adhesive was used. In the graphs obtained by using the Araldite 2011 and Araldite 2021-1 epoxy adhesives, there was a 24.4% increase in the maximum tensile force and a 26.2% increase in elongation at break compared to the graphs obtained by using the Araldite 2015-1 epoxy adhesive (Figure 14b). As a result of the tensile tests of the SLJ test specimens produced by bonding the ABS plates using three different epoxy adhesives by applying the 80 °C-1 h curing method, the maximum tensile force values varied between 1386.26 and 1743.98 N and the elongation at break values varied between 2.463 and 3.485 mm. As a result of the tensile tests of the SLJ test specimens produced by bonding the PLA plates using three different epoxy adhesives by applying the 80 °C-1 h curing method, the maximum tensile force values varied between 2690.48 and 3374.77 N and the elongation at break values varied between 3.260 and 4.300 mm. There are studies in the literature in which similar strengths were obtained by using different adhesives in PLA materials [14]. As a result, the three different epoxy adhesives used in the study showed much higher adhesion strength on the 3D-printed PLA plates than on the 3D-printed ABS plates. Additionally, no separation was observed between the layers of the 3D-printed PLA plates during the tensile tests. Therefore, it can be said that the interlayer shear strength of the 3D-printed PLA plates is much higher than the interlayer shear strength of the 3D-printed ABS plates. The highest adhesion strength and elongation at break values for both the 3D-printed ABS and PLA plates were obtained when the Araldite 2021-1 epoxy adhesive was used.

## 4. Conclusions

In this study, SLJ test specimens were produced by bonding 3D-printed ABS and PLA plates with three different two-component epoxy adhesives (Araldite 2011, Araldite 2015-1, and Araldite 2021-1) using five different curing methods (WO, 40 °C-3 h, 40 °C-16 h, 60 °C-2 h, and 80 °C-1 h). Tensile tests were applied to the produced SLJ test specimens. The effects of different epoxy adhesive types and different curing methods on the adhesion strength of the 3D-printed ABS and PLA plates were investigated.

The failure modes varied depending on the adhesive and curing method. The ABS SLJs bonded with Araldite 2011 exhibited AF failure across all curing methods, whereas the PLA SLJs showed a transition from AF to SBF failure at higher curing temperatures. For Araldite 2015-1, the ABS SLJs transitioned from AF to ACF failure at higher temperatures, while the PLA SLJs exhibited ACF failure in most conditions, except for SBF failure at 80 °C-1 h. The ABS SLJs bonded with Araldite 2021-1 displayed CF failure in the WO condition and SF failure under the remaining conditions, while the PLA SLJs bonded with the same adhesive consistently exhibited SBF failure.

The tensile test results indicated that the 80 °C-1 h curing method yielded the highest tensile force and elongation at break for the ABS and PLA plates, regardless of the adhesive used. The ABS SLJs bonded with Araldite 2011 and 2015-1 showed increased mechanical performance with higher curing temperatures, while Araldite 2021-1 demonstrated the lowest values under the WO condition. Similarly, the PLA SLJs bonded with Araldite 2021-1 exhibited the highest performance under the 80 °C-1 h curing method.

Under the 80 °C-1 h curing condition, the tensile force and elongation at break values for the ABS SLJs ranged from 1386.26 N to 1743.98 N and 2.463 mm to 3.485 mm, respectively, whereas the PLA SLJs exhibited values between 2690.48 N and 3374.77 N for tensile force and 3.260 mm and 4.300 mm for elongation at break. The findings highlight that the Araldite 2021-1 epoxy adhesive provided the highest adhesion strength and elongation at break for both the ABS and PLA plates.

## Figures and Tables

**Table 1:** The 3D printing parameters.

Material	Ultimaker ABS Orange	Ultimaker PLA Pearl White
Nozzle temperature	240 °C	210 °C
Bed temperature	85 °C	60 °C
Build volume temperature	36 °C	28 °C
Filament diameter	2.85 mm	
CAM program	Cura v4.8.0	
3D printer	Ultimaker S5	
Print speed	60 mm/s	
Travel speed	120 mm/s	
Nozzle diameter	0.4 mm	
Line width	0.4 mm	
Layer thickness	0.2 mm	
Wall thickness	0 mm	
Infill density	100%	
Infill pattern	Zig Zag	
Build plate adhesion type	None	

**Table 2:** Epoxy adhesive properties used in producing test specimens [29,35,36].

Adhesive Name	Araldite 2011	Araldite 2015-1	Araldite 2021-1
Tensile strength	24 MPa	31 MPa	42 MPa
Tensile modulus	1.9 GPa	1.6 GPa	1.8 GPa
Elongation at break	9%	4%	10%
Pot life at room temperature	100 min	45 min	3 min
Fixture time at room temperature	7 h	4 h	9 min

**Table 3:** Combinations used in the production of test specimens.

Case Number	Adherend	Adhesive	Curing Method
1	ABS	Araldite 2011	WO
2			40 °C-3 h
3			40 °C-16 h
4			60 °C-2 h
5			80 °C-1 h
6		Araldite 2015-1	WO
7			40 °C-3 h
8			40 °C-16 h
9			60 °C-2 h
10			80 °C-1 h
11		Araldite 2021-1	WO
12			40 °C-3 h
13			40 °C-16 h
14			60 °C-2 h
15			80 °C-1 h
16	PLA	Araldite 2011	WO
17			40 °C-3 h
18			40 °C-16 h
19			60 °C-2 h
20			80 °C-1 h
21		Araldite 2015-1	WO
22			40 °C-3 h
23			40 °C-16 h
24			60 °C-2 h
25			80 °C-1 h
26		Araldite 2021-1	WO
27			40 °C-3 h
28			40 °C-16 h
29			60 °C-2 h
30			80 °C-1 h

**Table 4:** Bonding failure modes of ABS and PLA materials bonded with Araldite 2011.

Adherend	Curing Method	Failure Modes				
		AF	CF	ACF	SF	SBF
ABS	WO	✓				
	40 °C-3 h	✓				
	40 °C-16 h	✓				
	60 °C-2 h	✓				
	80 °C-1 h	✓				
PLA	WO	✓				
	40 °C-3 h	✓				
	40 °C-16 h	✓				
	60 °C-2 h					✓
	80 °C-1 h					✓

**Table 5:** Bonding failure modes of ABS and PLA materials bonded with Araldite 2015-1.

Adherend	Curing Method	Failure Modes				
		AF	CF	ACF	SF	SBF
ABS	WO	✓				
	40 °C-3 h	✓				
	40 °C-16 h	✓				
	60 °C-2 h			✓		
	80 °C-1 h			✓		
PLA	WO			✓		
	40 °C-3 h			✓		
	40 °C-16 h			✓		
	60 °C-2 h			✓		
	80 °C-1 h					✓

**Table 6:** Bonding failure modes of ABS and PLA materials bonded with Araldite 2021-1.

Adherend	Curing Method	Failure Modes				
		AF	CF	ACF	SF	SBF
ABS	WO		✓			
	40 °C-3 h				✓	
	40 °C-16 h				✓	
	60 °C-2 h				✓	
	80 °C-1 h				✓	
PLA	WO					✓
	40 °C-3 h					✓
	40 °C-16 h					✓
	60 °C-2 h					✓
	80 °C-1 h					✓

## Data Availability

The original contributions presented in this study are included in the article. Further inquiries can be directed to the corresponding author.
